# Mild Inhibition of Mitochondrial Complex I Activates Neuroprotective Mechanisms by Enhancing Glucose Uptake and Restoring Energy Homeostasis in Alzheimer's Disease Models

**DOI:** 10.1002/alz70855_104475

**Published:** 2025-12-24

**Authors:** Thi Kim Oanh Nguyen, Sergey A Trushin, Mark Ostroot, Oscar Ramirez‐Molina, Eugenia Trushina

**Affiliations:** ^1^ Department of Neurology, Mayo Clinic, Rochester, MN, USA; ^2^ Universidad de Concepcion, Concepcion, Chile; ^3^ Mayo Clinic, Rochester, MN, USA; ^4^ Department of Molecular Pharmacology and Experimental Therapeutics, Mayo Clinic, Rochester, MN, USA

## Abstract

**Background:**

Although novel treatments targeting amyloid‐beta (Aβ) have been recently approved by the FDA, there remains a critical need for alternative therapeutic strategies to delay the onset and progression of Alzheimer's disease (AD). Mitochondrial dysfunction and disrupted brain energy homeostasis are key hallmarks of early‐stage AD. Developing treatments that address these deficits could offer substantial benefits. We identified mitochondrial complex I (mtCI) as a druggable target and demonstrated that its mild inhibition using the small molecule CP2 improves brain energy homeostasis, restores synaptic and cognitive function, and activates neuroprotective signaling in AD mouse models. However, the mechanistic link between mtCI inhibition, glucose metabolism, and energy homeostasis remains unclear.

**Methods:**

Cellular energy metabolism was assessed in neuroblastoma SH‐SY5Y cells expressing mutant human APP protein (APPswe) and in control cells. Glycolytic function, mitochondrial energetics, and fatty acid β‐oxidation (FAO) were measured using a Seahorse Analyzer. Glucose transporter (GLUT) translocation to the cell surface was evaluated using flow cytometry and immunofluorescence staining. Protein expression changes were analyzed via Western blot, and glucose uptake was quantified with the Glucose Uptake‐Glo™ assay. Additionally, metabolic flux analysis using 13C‐labeled glucose and targeted metabolomics by mass spectrometry were performed to measure specific metabolites.

**Results:**

APPswe cells displayed significant reductions in glycolysis and spare respiratory capacity, indicative of impaired mitochondrial energy production under stress. These deficits were accompanied by reduced glucose uptake, which was partially compensated by an increase in FAO. At concentrations relevant to *in vivo* treatments, CP2 acutely enhanced GLUT translocation to the cell surface, increasing glucose uptake within one hour. Enhanced glucose utilization in OXPHOS machinery was confirmed by inhibition of PDH phosphorylation and increased conversion of pyruvate to acetyl‐CoA, fueling the TCA cycle and generating critical signaling metabolites essential for gene regulation. These mechanisms were mediated by AMPK.

**Conclusion:**

Our findings suggest that mild inhibition of mtCI activates multiple neuroprotective mechanisms, including improved glucose metabolism and energy homeostasis. These consistent results in human cells and AD mouse models highlight the translational potential of this therapeutic approach for AD.

